# Influence of Match Status on serve execution and technical–tactical behaviors in men’s performance volleyball

**DOI:** 10.3389/fpsyg.2026.1741654

**Published:** 2026-01-21

**Authors:** Rubén Maneiro, Alan Davila, Claudio A. Casal, Iyán Iván-Baragaño

**Affiliations:** 1 Department of Special Didactics, Faculty of Education and Sport Sciences, University of Vigo, Vigo, Spain; 2 University of Vigo, Vigo, Spain; 3 Catholic University of Valencia San Vicente Mártir, Valencia, Spain; 4 Faculty of Physical Activity and Sports Sciences, University of León, León, Spain

**Keywords:** Match Status, observational methodology, performance, serve execution, volleyball

## Abstract

**Introduction:**

The serve is one of the most important technical-tactical behaviors in men’s performance volleyball and, at the same time, one of the least studied. Therefore, the aim of this study was threefold: first, to analyze the usual practices of these actions; second, to examine the relationship between the variables considered using Match Status as the reference criterion; and finally, to determine, at a multivariate level, the influence of the predictor variables on Match Status.

**Method:**

A total of 3,530 serves from 22 matches of the Spanish Superliga Masculina 2 during the 2023-2024 season were recorded. An observational methodology was applied within the mixed methods paradigm, using an ad hoc instrument with a field format and category system, and coding the data with the Lince Plus software. At the bivariate level, the association between Match Status and the other variables was assessed through contingency tables and the chi-square statistic. At the multivariate level, two classification models (a decision tree and a multinomial logistic regression) were trained using Match Status as the dependent criterion.

**Results:**

The results revealed significant associations between Match Status and six variables: Serve After Pause, Serving Player, Set Phase, Type of Serve, Result of the Previous Serve, and Execution Zone. The decision tree model identified the Result of the Previous Serve as the criterion with the highest informational gain, followed by Serve After Pause and the Server itself, and showed that serves executed after regulatory interruptions (e.g., time-outs) increased the probability of obtaining a favorable outcome. The multinomial regression confirmed the significant influence of Set Phase (χ^2^ = 40.311; *p* < 0.001), Reception Zone (χ^2^ = 17.784; *p* = 0.023), Serve After Pause (χ^2^ = 173.907; *p* < 0.001), Result of the Previous Serve (χ^2^ = 16.802; *p* = 0.002), and Type of Serve (χ^2^ = 17.232; *p* = 0.002). Performing the serve after a time-out significantly increased the odds of being ahead [Exp(B) = 3.13], and reception in zone 4 was associated with greater success [Exp(B) = 10.80].

**Conclusions:**

The conclusions highlight that serve effectiveness is multifactorial and provide practical applications for coaches to adjust serving and receiving strategies, considering match score dynamics and critical moments of the match.

## 1 Introduction

Volleyball is a team sport characterized by cooperation and opposition, divided space, and alternating participation, in which two teams of six players face each other on a court divided by a net. Each rally begins with a serve—an action that not only initiates the exchange but can also condition the opponent’s defensive and offensive structure. In this sense, the serve acts as the first tactical gesture of each rally, directly influencing the subsequent organization of play (Přidal et al., 2021).

In modern volleyball analysis, a distinction is commonly made between the different “game complexes” [complex I (KI) and complex II (KII)]. In the first, reception is decisive for offensive construction; in the second, the serve acquires crucial tactical relevance, as it aims to destabilize the opponent’s reception and limit the setter’s options (Palao et al., 2004; González et al., 2001).

In performance volleyball, the serve represents a fundamental technical–tactical component, as empirical evidence indicates that teams with greater effectiveness and proficiency in this action significantly increase their chances of victory (Cavedon et al., 2024; Sousa et al., 2023).

In recent years, elite players have adopted variants such as the Jump Serve and the Float Serve, not only for power but also as strategic tools to create uncertainty and force reception errors. Recent studies clearly reflect the differences between both modalities (Pawlik et al., 2024; Reiser and Depra, 2020). Jump serves tend to cause more reception errors and direct aces, although they also involve a higher risk of self-error (Sheppard et al., 2009). In contrast, Float Serves, less powerful but more unpredictable, are effective in disrupting the stability of the opponent’s reception system (Reiser and Depra, 2020). With regard to the preferred serving zone, the back–center zone was the most frequently targeted, followed by the back–left zone (zones 6 and 5, respectively). Furthermore, receptions performed with two players achieved better results than those with three (Ciuffarella et al., 2013), and the use of the forearms (underhand) for reception was more common when receiving fast serves directed to zone 1 (Paulo and Zaal, 2016).

In the study by Fatahi et al. (2022), it is demonstrated that the serve cannot be understood in isolation, but rather as a dynamic interaction between the Server and the receiver. In their study, they analyzed 204,165 serve receptions and found that the percentage of winning serves was very similar across the three Reception Zones (central, right, and left). Furthermore, in the study by Marcelino et al. (2011), the close relationship between Match Status and Type of Serve is also demonstrated, showing that teams leading on the scoreboard tend to take more risks with their serves, seeking to extend their advantage through more aggressive serves, whereas in high-pressure situations, teams tend to prioritize safety to avoid errors.

From a gender perspective, Kitsiou et al. (2020) found that men predominantly use the power Jump Serve, whereas women tend to use the Float Jump Serve. Moreover, serving in men’s volleyball shows greater spatial variability and higher risk when aiming at deep or difficult lateral zones (zones 5, 7, and 8) compared to women’s volleyball (zone 6). They also found that male competitors generally committed more errors, regardless of the Type of Serve or the chosen zone, than female players.

Physical factors cannot be overlooked either, as recent studies (Pawlik et al., 2024; Bari et al., 2023) indicate that upper and lower limb strength, jump power, and trunk stability are directly related to the speed and effectiveness of the serve, both in youth and professional players.

In conclusion, current research supports the view that the serve in volleyball is a complex action in which biomechanics, cognition, and tactics converge. Furthermore, it holds significant success. Its analysis should be approached comprehensively, considering not only the tactical aspects of the game and the current match score but also the process of motor counter-communication with the opponent.

Although scientific literature has advanced understanding of this action, available studies remain scarce. Although the scientific literature has advanced the understanding of this action, research remains limited, particularly regarding the influence of match status on serve execution. Therefore, the objectives of this study can be summarized in three points: first, to determine the usual serving practices in performance volleyball; second, through a bivariate analysis (accompanied by a chi-square test), to examine the relationship between the variables considered, using Match Status as the dependent criterion; and third, to determine, at a multivariate level, the influence of the predictor variables on Match Status.

## 2 Materials and methods

For this study, the observational methodology (Anguera, 1979) was applied, recognized as one of the most appropriate approaches for analyzing spontaneous behavior and interactions among athletes. This approach is also framed within the perspective of mixed methods, which combines qualitative and quantitative strategies (Anguera et al., 2014; Anguera and Hernández-Mendo, 2016).

Ethical review and approval were not required for the study on human participants in accordance with the local legislation and institutional requirements. Written informed consent from the [patients’/participants’ OR patients’/participants’ legal guardian/next of kin] was not required to participate in this study in accordance with the national legislation and the institutional requirements.

### 2.1 Design

The research design was nomothetic, as it focused on the analysis of multiple units of observation. Regarding the temporal dimension, a punctual design was adopted, since the study focused on a specific competition. Likewise, it is considered multidimensional, as it encompasses various levels of response (Anguera et al., 2011). This design falls within the third quadrant of those proposed by Anguera et al. (2011). The observation adhered to the principles of scientific rigor, ensuring full perceptibility in the recording of behaviors.

### 2.2 Participants

The sample selection was conducted using intentional or convenience observational sampling (Anguera et al., 2011). A total of 3,530 serving actions in volleyball were analyzed, recorded from 22 matches corresponding to the 2023–2024 season of the Spanish Superliga Masculina 2 championship.

The Superliga Masculina 2 volleyball competition during the 2023–2024 season was structured into two distinct phases: a regular phase (group stage) and a final phase or promotion play-off. The regular phase consisted of a group-based league format in which teams played home-and-away matches to determine the final standings. In Group A, the regular phase included matches between the following teams: Boiro Voleibol, Palencia, Miajadas, Gijón, Valladolid, Soria, Vigo, Dumbría, Textil Santanderina, Arona, San Sadurniño, and Almendralejo. All matches analyzed corresponding to this phase were played during the 2023–2024 regular season. After the conclusion of the regular phase, the top-ranked teams from each group qualified for the final phase, or promotion play-off, held at a single venue. This final phase was contested using a round-robin format among the six qualified teams to determine the promotion places to the Superliga Masculina. The final phase included matches between CV Textil Santanderina, Arona Playa de los Cristianos, Barça Voley, Servigroup Playas de Benidorm, UC3M Voleibol Leganés, and the second-ranked team from Group B. The matches analyzed in the present study correspond exclusively to the regular phase (group stage) of the Superliga Masculina 2 2023–2024 season. The competition structure comprises a group-based regular phase and a final promotion play-off held at a single venue among the top-ranked teams. The matches included in this study belong to the Group A regular phase, while matches from the final phase were not considered in the analysis.

During the 2023–2024 regular season of the Superliga Masculina 2, matches were played in a double-round-robin format (home-and-away) within each group, so each team faced every other team in its group twice (once at home and once away). No matches during the regular phase were held at neutral venues.

### 2.3 Registration and coding

The recording and concordance analysis process (Hernández-Mendo et al., 2014) was carried out using the Lince Plus software (Soto et al., 2019). Prior to the final coding, 10 observation sessions were conducted in accordance with the methodological guidelines proposed by Anguera et al. (2018, 1999). The recording was performed by a volleyball expert with over 10 years of experience and a degree in Physical Activity and Sports Sciences, who had previously completed 5 training sessions of 2 h each, in accordance with recommendations for acquiring observational competencies (Anguera et al., 2017). In the final training session, the intra-observer concordance coefficient (Cohen’s Kappa) was calculated using 10% of the total analyzed actions (*n* = 353) as the sample. Data quality control was verified using IBM SPSS Statistics version 25.0, yielding a Kappa value of 0.92, which is considered excellent according to the Fleiss et al. (2003) scale.

The recorded data correspond to level IV, that is, concurrent time-based data (Bakeman, 1978), indicating the presence of behavioral co-occurrences among players during the observed actions.

### 2.4 Observation instrument

To achieve the objectives of the study, an *ad hoc* instrument was designed that combines a field format with a category system, as proposed by Anguera et al. (2018). The proposed observation instrument can be consulted in Table 1.

**Table 1:** Observation instrument.

Criterion	Categories	Definition
Type of serve	(1) Float Serve from the Ground	(1) The ball is hit without spin from a stationary position.
	(2) Jump Float Serve	(2) Similar to the ground Float Serve, but with a short run-up and a small jump to increase power and angle.
	(3) Jump Serve	(3) The ball is tossed high, the player runs to gain momentum, and strikes it forcefully in the air, imparting spin and speed—similar to a spike.
Set phase	(1) Initial Points (0–10)	(1) Points between the first rally of the set and the point at which a team reaches 10. In the tie-break, this corresponds to points 0–7.
	(2) Intermediate Points (10–20)	(2) Points between the serve following point 10 and the serve for point 20. In the tie-break, this corresponds to points 7–12.
	(3) Critical Points (20–25)	(3) Final points of the set, between the serve for point 20 and the end of the set. In the tie-break, from point 12 onward.
Execution zone 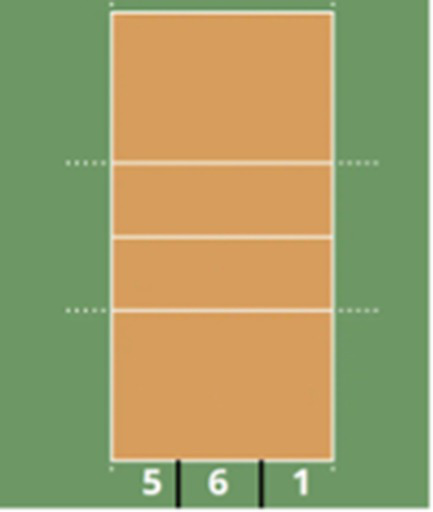	(1) Zone 1	(1) The player serves from the right–back area of the court.
	(2) Zone 6	(2) The player serves from the central–back area of the court.
	(3) Zone 5	(3) The player serves from the left–back area of the court.
Reception zone 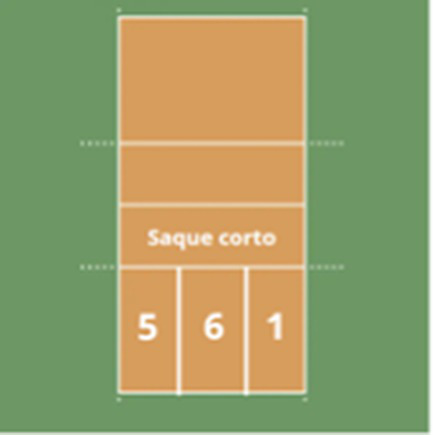	(1) Zone 1	(1) The opponent’s serve lands in area “1.”
	(2) Zone 6	(2) The opponent’s serve lands in area “6.”
	(3) Zone 5	(3) The opponent’s serve lands in area “5.”
	(4) Short Serve	(4) The opponent’s serve lands in the area defined as “Short Serve.”
	(5) Out	(5) Serve error.
Player executing the serve	(1) Setter	(1) Responsible for distributing play (2nd touch), setting the ball for attackers.
	(2) Receiver	(2) Player who combines reception and attack, positioned in the front–left and middle–back zones. Key in serve reception, defense, and wing attacks.
	(3) Opposite	(3) Usually, the team’s main attacker, located on the right side with the setter. Characterized by attacking power.
	(4) Middle Blocker	(4) Positioned in the front-center zone; specialist in blocking and quick attacks. Fundamental in defending against opponent attacks.
Receiving player	(1) Receiver	(1) Player who combines reception and attack, positioned in the front–left and middle–back zones. Key in reception, defense, and wing attacks.
	(2) Libero	(2) The team’s best receiver and defender, skilled in these areas, and characterized by the fact that they cannot attack or serve. Replaces middle blockers in the back row and wears a jersey of a different color.
	(3) Other	(3) Reception is performed by a player other than the libero or receiver.
	(4) None	(4) Serve error—no reception occurs.
Serve outcome 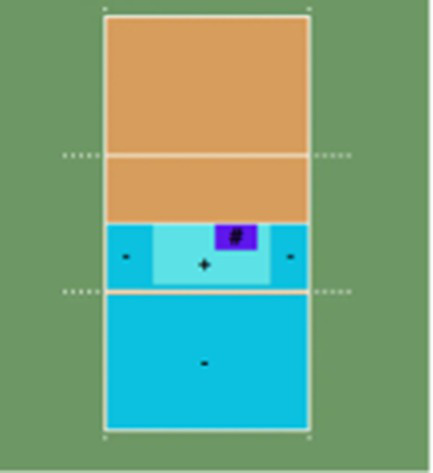	(1) Ace	(1) Direct point from the serve, either because the opponent fails to receive it or the ball lands directly inside the court.
	(2) Error	(2) Serve fault.
	(3) Double Positive Reception	(3) Perfect reception of the opponent’s serve; high-quality pass landing in the designated “#” zone.
	(4) Positive Reception	(4) Good reception that allows the setter to use all attackers, though not perfect. High and accurate pass landing in the “+” zone.
	(5) Negative Reception	(5) Poor reception prevents a quick or effective attack, or losing the quick option with the middle blocker. Represented by “-.”
	(6) Overpass	(6) Reception sent directly over the net into the opponent’s court.
Match Status	(1) Winning	(1) The serving team is ahead on the scoreboard.
	(2) Losing	(2) The receiving team is ahead on the scoreboard.
	(3) Drawing	(3) Both teams are tied on the scoreboard.
Serve After Pause	(1) Serve After Mop	(1) Serve following a short pause to dry the court or after a substitution, as both involve similar durations.
	(2) Serve After Time-Out	(2) Serve following a coach-called time-out to provide tactical instructions, correct errors, or interrupt the opponent’s momentum.
	(3) First Serve of the Set	(3) The team’s first serve at the beginning of the set.
	(4) No Pause	(4) Serve performed without any interruption.
Receiving team rotation	(1) 1	(1) The setter of the receiving team is in zone 1.
	(2) 2	(2) The setter of the receiving team is in zone 2.
	(3) 3	(3) The setter of the receiving team is in zone 3.
	(4) 4	(4) The setter of the receiving team is in zone 4.
	(5) 5	(5) The setter of the receiving team is in zone 5.
	(6) 6	(6) The setter of the receiving team is in zone 6.
Previous serve	(1) In	(1) The Previous Serve by your team landed in the court.
	(2) Error	(2) The Previous Serve by your team was a fault.
	(3) First Serve	(3) The first serve performed by each team in every set.

### 2.5 Data analysis

First, a descriptive (absolute and relative frequencies) and bivariate analysis was conducted using match status (winning, drawing, and losing) as the dependent criterion. The level of significance was set by default at *p* < 0.05. The degree of association was assessed using the chi-square statistic, and the effect size (ES) was evaluated through the contingency coefficient, considered small (ES = 0.10), medium (ES = 0.30), and large (ES = 0.50) (Gravetter and Wallnau, 2007). At the multivariate level, two multinomial classification models (decision tree and multinomial logistic regression) were trained using Match Status as the dependent criterion. The decision to employ both models is justified by the increased interpretability of the object of study. All analyses were performed using IBM SPSS Statistics version 25.0.

## 3 Results

As listed in Table 2, the majority of the serves were performed by the receiver (36.1%), in situations where the team was winning (54.3%), and were mainly executed without a pause (81.8%). Regarding the outcome of the serve, the most frequent category was negative reception (28.9%), and the most common serving player was the receiver (60.0%).

**Table 2:** Descriptive results.

Criterion		Frequency	Percentage (%)
Player executing the serve	Setter	612	17.3
	Receiver	1,275	36.1
	Opposite	544	15.4
	Middle Blocker	1,099	31.1
Match status at the moment of the serve	Winning	1,916	54.3
	Drawing	1,128	32.0
	Losing	486	13.8
Serve after pause	Serve after mop (court drying)	343	9.7
	Serve after time-out	220	6.2
	First Serve of the Set	80	2.3
	No pause	2,887	81.8
Serve outcome	Ace	181	5.1
	Error	466	13.2
	Double positive reception	832	23.6
	Positive reception	878	24.9
	Negative reception	1,019	28.9
	Overpass	154	4.4
Receiving player	Receiver	2,119	60.0
	Libero	897	25.4
	Others	49	1.4
	None	465	13.2
Set phase	Initial points (0–10)	1,384	39.2
	Intermediate points (11–20)	1,401	39.7
	Critical points (21–30)	745	21.1
Type of serve	Float Serve from the ground	22	0.6
	Jump Float Serve	2,256	63.9
	Jump Serve	1,252	35.5
Receiving team rotation	1	641	18.2
	2	528	15.0
	3	544	15.4
	4	541	15.3
	5	618	17.5
	6	658	18.6
Previous serve	In	2,960	83.9
	Error	410	11.6
	First serve	160	4.5
Execution zone	Zone 1	1,482	42.0
	Zone 6	766	21.7
	Zone 5	1,282	36.3
Reception zone	Zone 1	769	21.8
	Zone 6	1,266	35.9
	Zone 5	957	27.1
	Short Serve	74	2.1
	Out	464	13.1

With respect to the set progression, points were mainly concentrated in the intermediate phase (39.7%), with Jump Float Serves (63.9%) being the most frequent type. Rotation 6 of the receiving team was the most used (18.6%), the Previous Serve was in (83.9%), and Execution Zone 1 (42.0%) and Reception Zone 6 (35.9%) were the most common during play.

At the bivariate level, and to determine the relationship between the criterion “Match Status” and the other criteria considered, a contingency table with a chi-square test was constructed to compare the level of effectiveness achieved across the different criteria included in the observation instrument. The results are shown in Table 3.

**Table 3:** Bivariate results, with the criterion “Match Status” as the reference criterion.

Criteria	Categories	Winning	Drawing	Losing	χ^2^	*p*-value	Contingency coefficient
Player executing the serve	Setter	337 (55.1%)	174 (28.4%)	101 (16.5%)	11.42	0.07	0.05
	Receiver	713 (55.9%)	391 (30.7%)	171 (13.4%)			
	Opposite	285 (52.4%)	189 (34.7%)	70 (12.9%)			
	Middle Blocker	581 (52.9%)	374 (34%)	144 (13.1%)			
Serve After Pause	After mopping	198 (57.7%)	116 (33.8%)	29 (8.5%)	532.0	<0.001	0.388
	After time-out	160 (72.7%)	50 (22.7%)	10 (4.5%)			
	First Serve of the Set	1 (1.3%)	0 (0%)	79 (98.8%)			
	No pause	1,567 (53.9%)	397 (12.7%)	922 (33.3%)			
Serve outcome	Ace	109 (60.2%)	48 (26.5%)	24 (13.3%)	16.8	0.07	0.06
	Error	248 (53.2%)	160 (34.3%)	58 (12.4%)			
	Double positive reception	438 (52.6%)	258 (31%)	136 (16.3%)			
	Positive reception	465 (53%)	297 (33.8%)	116 (13.2%)			
	Negative reception	561 (55.1%)	329 (32.3%)	129 (12.7%)			
	Overpass	95 (61.7%)	36 (23.4%)	23 (14.9)			
Receiving player	Receiver	1,124 (53%)	702 (33.1%)	293 (13.8%)	16.11	0.01	0.06
	Libero	520 (58%)	255 (28.4%)	122 (13.6%)			
	Other	25 (51%)	11 (22.4%)	13 (26.5%)			
	None	247 (53.1%)	160 (34.4%)	58 (12.5%)			
Set phase	Initial points (0–10)	736 (53.2%)	361 (54.4%)	287 (20.7%)	106.2	<0.001	0.17
	Intermediate points (11–20)	762 (54.4%)	503 (35.9%)	136 (9.7%)			
	Critical points (21–30)	418 (56.1%)	264 (35.4%)	63 (8.5%)			
Type of serve	Float Serve from the ground	12 (54.5%)	6 (27.3%)	4 (18.4)	20.36	<0.001	0.07
	Jump Float Serve	1,169 (51.8%)	779 (34.5%)	308 (13.7%)			
	Jump Serve	735 (58.7%)	343 (27.4%)	174 (13.9%)			
Receiving team rotation	1	361 (56.3%)	192 (30%)	88 (13.7%)	3.87	0.95	0.03
	2	289 (54.7%)	165 (31.3%)	74 (14%)			
	3	285 (52.4%)	183 (33.6%)	76 (14%)			
	4	288 (53.2%)	177 (32.7%)	76 (14%)			
	5	336 (54.4%)	205 (33.2%)	77 (12.5%)			
	6	357 (54.3%)	206 (31.3%)	95(14.4%)			
Previous serve	In	1,680 (56.8%)	941 (31.8%)	339 (11.5%)	283.1	<0.001	0.28
	Error	186 (45.4%)	168 (41%)	56 (13.7%)			
	First Serve	50 (31.3%)	19 (11.9%)	91 (56.9%)			
Execution zone	Zone 1	785 (53%)	478 (32.3%)	219 (14.8%)	12.38	0.01	0.05
	Zone 6	454 (59.3%)	213 (27.8%)	99 (12.9%)			
	Zone 5	677 (52.8%)	437 (34.1%)	168 (13.1%)			
Reception zone	Zone 1	412 (53.6%)	239 (31.1%)	118 (15.3%)	12.7.	0.12	0.06
	Zone 6	687 (54.3%)	399 (31.5%)	180 (14.2%)			
	Zone 5	528 (55.2%)	316 (33%)	113 (11.8%)			
	Short Serve	47 (63.5%)	24 (32.4%)	3 (4.1%)			
	Out	242 (52.2%)	150 (32.3%)	72 (15.5%)			

As observed, six variables presented a statistically significant relationship: Serve After Pause (χ^2^ = 532; *p* < 0.001), Receiving Player of the Serve (χ^2^ = 16.11; *p* = 0.01), Set Phase (χ^2^ = 106.2; *p* < 0.001), Type of Serve (χ^2^ = 20.63; *p* < 0.001), Previous Serve (χ^2^ = 238.1; *p* < 0.001), and Execution Zone (χ^2^ = 12.38; *p* = 0.01). Moreover, a clear trend was observed for the variables of the Player Performing the Serve (χ^2^ = 11.42; *p* = 0.07) and of the Serve Outcome (χ^2^ = 16.8; *p* = 0.07).

**Figure 1:**
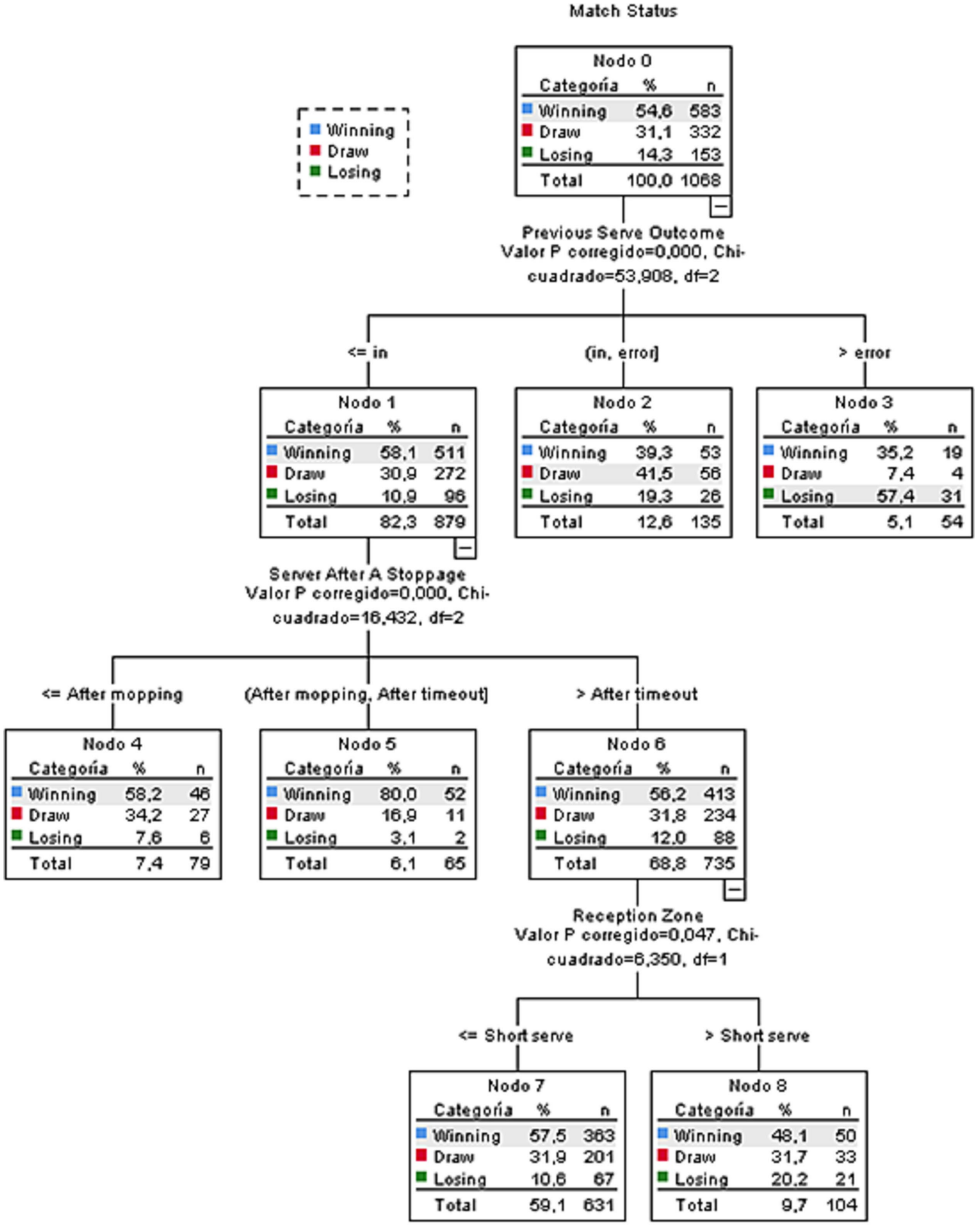
Decision tree model.

The results of the decision tree based on the CHAID algorithm are presented in Figure 1. The analysis identified combinations of variables that best explain the categories of Match Status (winning, drawing, or losing). This model comprised nine nodes, six of which were terminal. The theoretical results of the model are presented in Table 4.

**Table 4:** Summary of the presented model.

Model summary		
Specs	Growth method	CHAID
	Dependent criterion	Match Status
	Independent criteria	Type of Serve, Serving Zone, Reception Zone, Server, Serve Outcome, Server After a Stoppage, Server Receiver, Opponent Rotation, Previous Serve Outcome, Stage of the Set
	Validation	Sample division
	Maximum depth	3
	Minimum cases in the parent node	100
	Minimum cases in the child node	50
Results	Dependent criteria included	Previous Serve Outcome, Server After a Stoppage, Server
	Number of nodes	9
	Number of terminal nodes	6
	Depth	3

To carry out the validation process, the total sample of possessions (3,530) was divided into a testing sample (30%, *n* = 1,068) and a training sample (70%, *n* = 2,471). Next, the model’s predictive capacity is presented in Table 5 (classification table). In this way, the goodness of fit and the performance of the model can be evaluated (Table 6). The model, based on the chi-squared automatic interaction detection (CHAID) method and using Match Status as the dependent criterion, achieved a correct classification rate of 56.0% in the training set. In this set, the accuracy rates were 87.7% for predicting winning situations, 16.9% for drawing situations, and 20.3% for losing situations. In the validation phase (testing set), the overall classification accuracy was 54.5%, with correct predictions of 87.7% for winning, 14.1% for drawing, and 18.0% for losing serves (Table 5).

**Table 5:** Classification of the model.

Classification					
Sample	Observed	Predicted			
		Winning	Draw	Losing	Accuracy (%)
Training	Winning	511	53	19	87.7
	Draw	272	56	4	16.9
	Losing	96	26	31	20.3
	Percentage global	82.3%	12.6%	5.1%	56.0
Test	Winning	1,169	133	31	87.7
	Draw	669	112	15	14.1
	Losing	243	30	60	18.0
	Percentage global	84.5%	11.2%	4.3%	54.5

Growing methods: CHAID.

Dependent criterion: Match Status.

**Table 6:** Risk of the predictive model.

Risk		
Sample	Estimate	Typical error
Training	0.440	0.015
Contrast	0.455	0.010

Growing methods: CHAID.

Dependent criterion: Match Status.

As shown in Figure 1, the criterion with the greatest influence is Previous Serve Outcome, which is the root node of the model. This result indicates that the immediately preceding dynamics within the sequence of play exert a determining influence on the probability of the subsequent Match Status. Specifically, when the Previous Serve results in a direct point or an opponent’s error, the likelihood of being in a favorable situation increases, whereas Previous Serves with a neutral result or self-error are associated with higher chances of drawing or losing.

Following this first partition, the criterion with the next highest information gain is Server After a Stoppage. In nodes where the Previous Serve was unsuccessful, performing the next Serve After a Stoppage (whether due to a time-out, substitution, or interruption of play) increases the probability of reversing an unfavorable situation and achieving a balanced or positive score.

At a third level, the criterion Server (the Player Performing the Serve) also contributes to relevant information gain, introducing differences among the terminal nodes. This result suggests that the influence of the tactical context is modulated by the player’s individual characteristics, including technical level, risk profile, and ability to maintain performance under pressure.

The multinomial logistic regression analysis presented in Table 7 included only the previously significant independent criteria (Stage of the Set, Reception Zone, Server After a Stoppage, Previous Serve Outcome, and Type of Serve), and categories with fewer than 5% of cases were removed to eliminate potential outliers that could influence the model.

**Table 7:** Results of the multinomial logistic regression analysis.

Parameter estimation									
Match Status^a^		B	Standard error	Wald	df	Significance	Exp(B)	Exp(B) (95% CI)	
								Lower limit	Upper limit
Winning	Intercept	1,844	0.393	21,989	1	0.000			
	[Stage of the Set = 1]	−0.563	0.162	12,145	1	0.000	0.569	0.415	0.782
	[Stage of the Set = 2]	−0.151	0.166	0.827	1	0.363	0.860	0.622	1,190
	[Stage of the Set = 3]	0^b^			0				
	[Reception Zone = 1]	0.097	0.182	0.281	1	0.596	1,102	0.770	1,575
	[Reception Zone = 2]	0.210	0.,17	1,525	1	0.217	1,234	0.884	1,721
	[Reception Zone = 3]	0.354	0.182	3,788	1	0.052	1,425	0.998	2,035
	[Reception Zone = 4]	2,380	0.895	7,067	1	0.008	10,803	1,869	62,447
	[Reception Zone = 5]	0^b^			0				
	[Server After A Stoppage = 1]	0.355	0.212	2,803	1	0.094	1,426	0.941	2,161
	[Server After A Stoppage = 2]	1.141	0.334	11,640	1	0.001	3,129	1,625	6,026
	[Server After A Stoppage = 3]	−5,908	1,072	30,349	1	0.000	0.003	0.000	0.022
	[Server After A Stoppage = 4]	0^b^			0				
	[Previous Serve Outcome = 1]	−0.143	0.335	0.182	1	0.669	0.867	0.450	1,670
	[Previous Serve Outcome = 2]	−0.494	0.363	1,853	1	0.173	0.610	0.299	1,243
	[Previous Serve Outcome = 3]	0^b^			0				
	[Type of Serve = 1]	−0.259	0.657	0.156	1	0.693	0.771	0.213	2,796
	[Type of Serve = 2]	−0.171	0.116	2,176	1	0.140	0.843	0.672	1,058
	[Type of Serve = 3]	0^b^			0				
Draw	Intercept	1,015	0.440	5,334	1	0.021			
	[Stage of the set = 1]	−0.830	0.170	23,812	1	0.000	0.436	0.313	0.609
	[Stage of the set = 2]	−0.107	0.171	0.389	1	0.533	0.899	0.642	1,257
	[Stage of the Set = 3]	0^b^			0				
	[Reception Zone = 1]	0.014	0.194	0.005	1	0.942	1,014	0.693	1,484
	[Reception Zone = 2]	0.106	0.181	0.343	1	0.558	1,112	0.780	1,583
	[Reception Zone = 3]	0.309	0.192	2,588	1	0.108	1,362	0.935	1,986
	[Reception Zone = 4]	2,201	0.911	5,845	1	0.016	9,038	1,517	53,837
	[Reception Zone = 5]	0^b^			0				
	[Server After A Stoppage = 1]	0.179	0.222	0.651	1	0.420	1,196	0.774	1,848
	[Server After A Stoppage = 2]	0.426	0.356	1,439	1	0.230	1,532	0.763	3,075
	[Server After A Stoppage = 3]	−23,918	0.000		1		4,097E-11	4,097E-11	4,097E-11
	[Server After A Stoppage = 4]	0^b^			0				
	[Previous Serve Outcome = 1]	0.101	0.382	0.070	1	0.791	1,107	0.524	2,338
	[Previous Serve Outcome = 2]	0.188	0.407	0.213	1	0.645	1,206	0.543	2,679
	[Previous Serve Outcome = 3]	0^b^			0				
	[Type of Serve = 1]	−0.164	0.720	0.052	1	0.819	0.848	0.207	3,482
	[Type of Serve = 2]	0.161	0.124	1,694	1	0.193	1,175	0.922	1,499
	[Type of Serve = 3]	0^b^			0				

^a^Reference category.

^b^This parameter is set to zero because it is the reference category.

The likelihood ratio tests indicated that Stage of the Set (χ^2^ = 40.311, *p* < 0.001), Reception Zone (χ^2^ = 17.784, *p* = 0.023), Server After a Stoppage (χ^2^ = 173.907, *p* < 0.001), Previous Serve Outcome (χ^2^ = 16.802, *p* = 0.002), and Type of Serve (χ^2^ = 17.232, *p* = 0.002) contributed significantly to the multinomial model.

The results showed that the Set Phase and Reception Zone were significantly associated with Match Status. For example, early points reduced the probability of winning compared to losing (B = −0.563, *p* < 0.001, Exp[B] = 0.569, 95% confidence interval [CI]: 0.415–0.782), whereas receiving in zone 4 increased this probability (B = 2.380, *p* = 0.008, Exp[B] = 10.803, 95% CI: 1.869–62.447). Likewise, serving after a time-out increased the likelihood of winning (B = 1.141, *p* = 0.001, Exp[B] = 3.129, 95% CI: 1.625–6.026). Other factors, such as the Result of the Previous Serve and the Type of Serve, showed smaller but directionally consistent effects.

The model presented a significant overall fit (−2 log likelihood = 821.284, χ^2^ = 450.479, df = 26, *p* < 0.001) and a Nagelkerke pseudo-R^2^ = 0.140, indicating that, although it does not explain the total variability in Match Status, it significantly captures the relationship between game characteristics and the probability of winning, losing, or drawing.

## 4 Discussion

The present study was designed with a threefold objective: first, at the univariate level, to identify the usual execution practices of these actions in performance volleyball; second, at the bivariate level, to examine the relationship between the variables considered, using Match Status as the reference criterion; and third, at the multivariate level, to determine the influence of the predictor variables on Match Status.

With regard to the first objective, the univariate results show a clear predominance of the Jump Float Serve (63.9%), reflecting a preference for a Type of Serve that effectively balances efficiency and error control in high-level competitions. This finding aligns with the observations of Silva et al. (2014), who emphasize that the serve as one of the key performance-determining skills, capable of generating direct points or conditioning the development of the rally depending on its accuracy and power.

In contrast, the high proportion of negative receptions (28.9%) is consistent with the review by Palao et al. (2004), which highlights the importance of serve reception as a decisive action in the construction of offensive play and, consequently, a critical factor in collective performance. The difficulty in controlling aggressive or float-effect serves may explain the observed frequency of ineffective receptions, reinforcing the need for specialized technical training in this phase.

With regard to the player receiving the serve, the study by João et al. (2006), which analyzed receptions from a group in the 2001 World League, reported similar data regarding the percentage of receptions made by the libero and the receivers—33.8 and 66.2%, respectively. Lirola and González (2009) also indicated that, in the 2001 Volleyball World League, the libero performed 36.4% of the receptions, while the two receivers accounted for 63.6%, corresponding individually to 31.8%—figures very similar to those obtained in this study. In contrast to these findings, the study by Arias et al. (2011), conducted with youth players, showed that the players categorized as “others” performed the highest percentage of receptions, surpassing both receivers and the libero. These results may be due to the fact that serving players often try to avoid directing the serve toward the defensive specialist (the libero), who is also typically an excellent receiver (Arias et al., 2011).

Concerning the second objective, the six variables identified as significant (Serve After Pause, Receiving Player, Set Phase, Type of Serve, Previous Serve Outcome, and Execution Zone) demonstrate that serve performance does not depend solely on individual technique but also on the tactical and situational context in which each action occurs. The significant association between Match Status and Serve After Pause (χ^2^ = 532, *p* < 0.001) suggests that teams use time-outs and other interruptions as tactical tools to modify the game’s dynamics. This behavior can be interpreted as a strategy to break negative momentum or reinforce the Server’s focus during critical moments of the set. The scientific literature has described that tactical pauses can influence competitive momentum, temporarily affecting the effectiveness of subsequent actions, as observed in the case of serves performed immediately after play resumes (Fernández-Echeverría et al., 2019).

Likewise, the association between the Type of Serve and the Match Status is consistent with the findings of Silva et al. (2014), who indicate that the choice of serve type reflects a balance between risk and reward. More aggressive serves, such as those executed with a jump or greater power, may increase the likelihood of error but also place greater pressure on the opponent’s reception and enhance the chances of scoring direct points or limiting the quality of the opponent’s response. This pattern appears to be reflected in the observed data, in which the Type of Serve varies with the match situation.

Furthermore, the relationship between the serve Execution Zone and Match Status (χ^2^ = 12.38, *p* = 0.01) aligns with the findings of Arias et al. (2011), who emphasize that the selection of serve direction or target zone constitutes a fundamental tactical element. These authors highlight that directing the serve toward specific zones can increase the difficulty of the opponent’s reception and affect the quality of the first contact, particularly during critical moments of the match.

Regarding the third objective, the combined analysis of results obtained from the decision tree and the multinomial logistic regression provides a more comprehensive understanding of the factors that explain variations in Match Status during play. Both techniques, although differing in approach, converge in highlighting the influence of contextual aspects such as the outcome of the Previous Serve, the occurrence of pauses or time-outs, the Type of Serve, the Reception Zone, and the phase of the set.

In the case of the decision tree, the criterion with the greatest information gain was the Previous Serve Outcome, followed by Serve After Pause and the player executing the serve. This order suggests that both the immediate sequence of play and tactical interruptions play a decisive role in the subsequent evolution of the score. It was observed that serves performed after a pause tend to be associated with improved performance and a higher probability of reversing unfavorable situations. These results are consistent with those obtained by Fernández-Echeverría et al. (2019), who found that time-outs can alter competitive dynamics and promote a more effective response upon resumption of play.

The multinomial logistic regression, in turn, confirmed the importance of the same variables identified in the decision tree but provided a quantitative estimation of their effects. The model showed that performing a Serve After Time-Out significantly increases the likelihood of being in a winning situation, while receiving in zone four enhances the chances of success. This result is consistent with the findings of Paulo and Zaal (2016), who indicated that the receiver’s position and Reception Zone influence the effectiveness of the action, and with the observations of Silva et al. (2014), who emphasized that serving and receiving are two of the most influential actions in achieving victory. Furthermore, the influence of Match Status and Set Phase on technical execution aligns with what was Marcelino et al. (2011) reported: players adjust their behavior according to the score and the opponent’s level.

The results obtained expand the existing evidence by clearly quantifying the influence of serving after a pause and the Reception Zone on the probability of success. Nonetheless, the width of some confidence intervals—particularly for the zone four criterion—suggests that the observed magnitudes should be interpreted with caution and that further analyses with larger samples are needed. In any case, the dataset as a whole confirms that serving performance depends not only on technical execution but also on the tactical context and competitive moment, as previously noted by Palao et al. (2004), and by Hileno et al. (2020), who highlighted the relevance of the sequencing of actions within the development of the rally.

## 5 Conclusion

The main conclusion of the present study are as follows. First, it was confirmed that the effectiveness of the serve does not depend solely on the player’s technical skill but also on the context in which it is executed. Factors such as the Previous Serve Outcome, the existence of pauses or time-outs, the phase of the set, and the Reception Zone showed a significant relationship with the evolution of the score, underscoring the complex and multifactorial nature of this action. Second, serves performed after a pause or time-out tended to be associated with a higher probability of success, suggesting that these interruptions may serve as tactical resources to modify the rhythm of play and enhance performance. Third, receiving in zone four was linked to a greater likelihood of achieving a favorable outcome, reinforcing the tactical importance of serve direction and reception positioning. Finally, the results confirm that the serve should not be understood as an isolated technical action but rather as part of a broader tactical framework that responds to the specific moment of the match and the dynamic conditions of the game.

### 5.1 Practical applications

From a practical perspective, coaches may design serving strategies according to Match Status, adjusting the level of risk associated with the serve (Type of Serve and Execution Zone) depending on whether the team is winning, drawing, or losing. In this regard, the findings indicate that serves executed after regulatory interruptions, particularly after time-outs, significantly increase the likelihood of achieving a favorable outcome. Therefore, these moments may be intentionally used to reinforce the Server’s concentration and to modify match dynamics.

In addition, directing the serve toward zones that hinder high-quality reception and constrain the organization of the opponent’s counterattack may be incorporated as a key tactical criterion, especially during intermediate and critical phases of the set. Complementarily, these results may assist coaches in optimizing pressure-based serve training by recreating realistic match scenarios involving stoppages, decisive points, and different score situations, as well as in adjusting reception systems according to the serving tendencies observed in the opponent.

### 5.2 Future lines of research

Future research should move toward an integrated analysis of the serve–reception–block sequence, to better understand how reception quality conditions counterattack development and how the blocking system influences the setter’s decision-making process. In this context, it would be relevant to examine the interaction among serve type, Execution Zone, reception quality, and block configuration, assessing their combined impact on subsequent offensive effectiveness.

Additionally, future studies may incorporate sequential or probabilistic analytical approaches to explore the temporal dynamics of these interactions within the rally and to compare these patterns across different competitive levels, age categories, and sexes. Finally, the inclusion of additional contextual variables, such as scoreboard pressure during critical points or the specific role of the Receiving Player, would contribute to a more comprehensive understanding of technical-tactical performance in volleyball.

## Data Availability

The raw data supporting the conclusions of this article will be made available by the authors, without undue reservation.
